# The Relationship of COVID-19 Vaccination Status with Health Literacy of Syrians Living in Istanbul

**DOI:** 10.3390/vaccines11091394

**Published:** 2023-08-22

**Authors:** Esmehan Aysit, Hatice Ikiisik, Mustafa Cakir, Isil Maral

**Affiliations:** Department of Public Health, Faculty of Medicine, Istanbul Medeniyet University, Istanbul 34700, Turkey; drhatice.ikiisik@gmail.com (H.I.); dr.mustafacakir34@gmail.com (M.C.); isilmrl@gmail.com (I.M.)

**Keywords:** COVID-19 vaccinations, refugee vaccinations, health literacy, global health, barriers against vaccination

## Abstract

Health literacy is an important determinant of health care use among refugees and migrant communities. This present study aimed to evaluate the relationship between health literacy levels, sociodemographic characteristics, and the status of receiving the COVID-19 vaccine in Syrians under “Temporary Protection” in Istanbul. This study was conducted in February, March, and May 2022 in an Extended Migrant Health Center in Istanbul, with a survey prepared in Arabic under observation. A total of 571 questionnaires were analyzed. The mean age of the participants was 31.92 ± 6.14, and 80.7% were female and 26.6% were high school graduates. A total of 55.0% of Syrians have not had any of the COVID-19 vaccines. The health literacy level of 1.1% of the immigrants was determined as “excellent”, 68.7% as “inadequate”, 20.7% as “problematic/limited”, and 9.6% as “adequate”. According to the logistic regression model, being male, of elder age, middle and above economic status, and having a chronic disease in the family were determined as the variables associated with the status of being vaccinated against COVID-19. Refugees are a group often exposed to inequalities in access to health services. Increasing health literacy in these groups will provide a significant improvement in access to and use of health services.

## 1. Introduction

Turkey has been exposed to an intense migration that started with the internal disturbance that emerged in Syria in 2011 and extended to the present day [1]. As a result, it has become the country hosting the greatest number of refugees in the world for the fifth consecutive year in 2020. The vast majority of refugees in Turkey are Syrian [2]. As of January 2023, the number of Syrians defined as “Temporary Protection” in the country has reached 3,522,000. With an immigrant population exceeding 540,000, the province with the highest immigrant population is Istanbul [3]. Immigrants can benefit from primary, secondary, and tertiary health services, including emergency health services, free of charge [1,4]. It is known that individuals’ access to and use of health services is related to both health literacy and individual health behaviors. Health literacy, which has an indisputable role in the effective use and efficiency of health services, is also effective in eliminating health inequalities [5,6]. Those with poor health literacy use preventive health services less, which poses a critical problem, especially concerning infectious and chronic diseases [7,8]. Immigrants are populations that experience the significant consequences of health literacy inadequacy. Immigrants with sociological, psychological, and economic difficulties in their new environments also do not know how, from where, and within the framework of which rules they can access health services, and language and communication problems are one of the critical leading obstacles [9,10].

The European Union has taken decisions to provide financial and social support to the Republic of Turkey due to the increasing Syrian migrant population in the country. The SIHHAT Project, financed by the European Union, under the Ministry of Health since 2016, is the most comprehensive and largest collaboration on migration management in the field of health, and is designed to gradually improve the existing health care offered to immigrants [1]. In addition to free emergency health services, free preventive health services and polyclinic services are provided to immigrants in Turkey through foreign national outpatient clinics as well as immigrant health and strengthened immigrant health centres. All services provided are at the same facilities attended by the local population. Health services are provided by Extended Migrant Health Centers (EMHC) in settlements with more than 20,000 Syrians. There are 36 centres affiliated to the Ministry of Health for immigrants in Istanbul, and only 8 of these centres are EMHC [11]. Once more, within the scope of the SIHHAT project, the health literacy levels of Syrians Under Temporary Protection were measured using the THL-32 (Turkish Health Literacy Scale-32) Scale in 2018 and 2020. As a result of the study conducted in 2018 (more than 80%) and as a result of the study conducted in 2020, more than 75% were determined to have insufficient and problematic/limited health literacy [12]. As a result of the study performed by the WHO (World Health Organization) on Syrians under Temporary Protection in Turkey in 2019 using the HLS-EU Q16 (European Health Literacy Survey Questionnaire) and S-FHL (Swedish Functional Health Literacy Scale), about half of them had insufficient and problematic/limited understanding for health literacy, and more than 80% of them had insufficient and problematic/limited functional health literacy [13].

The ongoing COVID-19 pandemic since 2019 has been a major global public health problem, with more than 750 million reported cases and more than 6.5 million deaths [14]. In this period, when the need for health services has increased, the need for health literacy has become more evident for immigrants. Refugees in Turkey have been granted free access to COVID-19 testing and treatment during the pandemic. Moreover, brochures containing pandemic prevention, control, and treatment information specific to this population were distributed in Arabic through media and mobile phone communication [15]. In addition to many measures taken throughout the country, vaccination studies have started as of January 2021 [16]. A cross-sectional study conducted with immigrants who applied to the Turkish Red Crescent Community Center in 15 provinces in the same year revealed that 71.1% of those under Temporary Protection were vaccinated with at least one dose of the COVID-19 vaccine [17]. As of January 2023, 85.69% of the population has received two vaccine doses in the country. No separate data were detected for immigrants in the Ministry of Health data [18]. Studies have been conducted on the acceptance of the COVID-19 vaccine by the local people of Turkey, and it has been revealed that factors such as gender, age, education level, occupation, and high-risk perceptions are effective in vaccine purchase [19].

Although there are studies conducted on immigrants during the pandemic, no study has been determined to evaluate the relationship between the COVID-19 vaccine and vaccination status and health literacy. This study aimed to evaluate the relationship between the health literacy levels and socio-demographic characteristics of those with the COVID-19 vaccine who applied to a large and comprehensive Extended Migrant Health Center in Istanbul, a city in the country with the most concentrated and ever increasing population of immigrants.

## 2. Materials and Methods

### 2.1. Design of the Study

This study was performed in the Extended Migrant Health Center in Istanbul, the province with the highest immigrant density in Turkey. The data were collected on Mondays, Wednesdays, and Fridays in February, March, and May 2022. The participants were surveyed under observation, paying attention to the COVID-19 precautions. The purpose of the study was explained to all Syrians Under Temporary Protection who applied to the center by an Arabic-speaking researcher. Although their legal status in Turkey was defined as “Syrian Under Temporary Protection”, they were included as “immigrants” in the article.

### 2.2. Individuals Included in the Study

**Figure 1:**
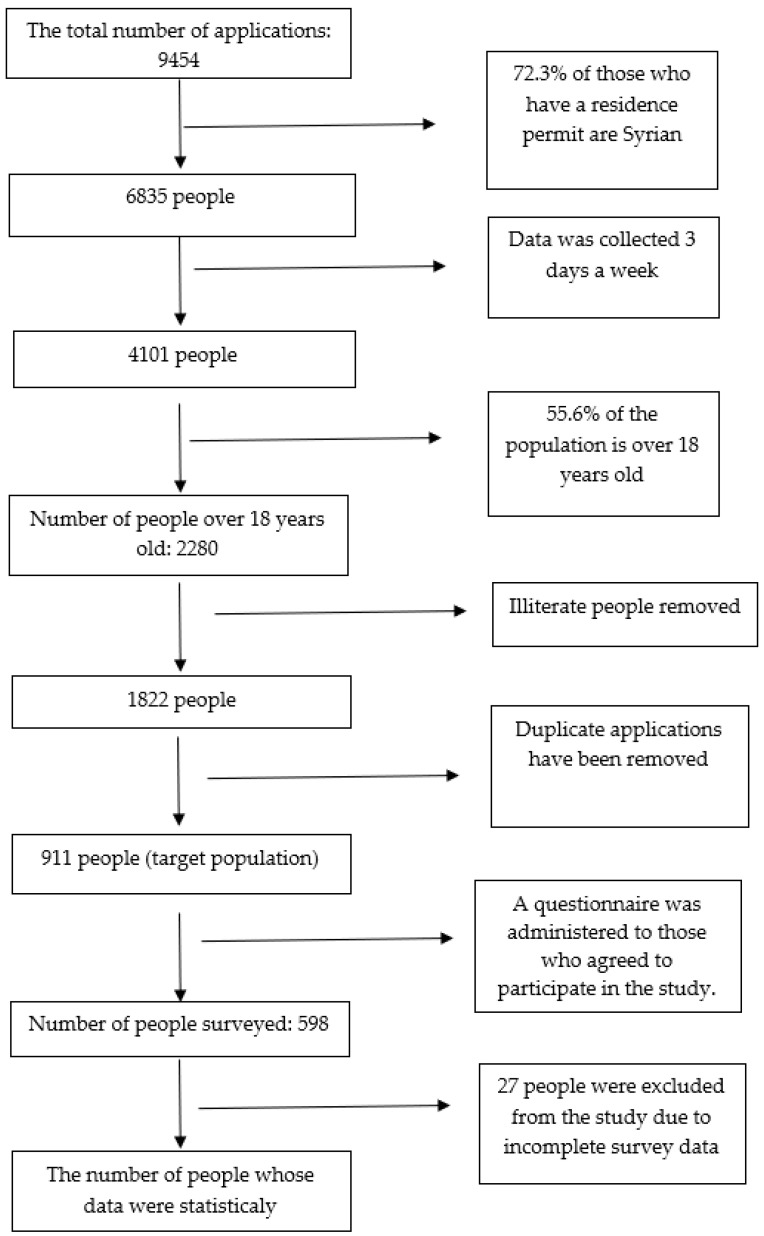
Individuals included in the study.

The study is a cross-sectional descriptive study. The total number of quarterly applications to the EMHC is 9454 (2802 in February, 3324 in March 2022, and 3328 people in May). In April 2022, there was a decrease in the number of applications, due to it being the holy month of Ramadan. These numbers consisted of both Syrian Under Temporary Protection and other immigrant applications. Of the applicants, 6385 were Syrian nationals (72.3% of foreign nationals with a residence permit are Syrians in 2022 [3]). The research was applied to Syrian nationals who applied to the center on Mondays, Wednesdays, and Fridays. If the same person applied more than once in these three days, then only their initial application was included in the research. Being literate and 18 years or older were the criteria for inclusion in the research. The percentage of Syrians over the age of 18 living in Turkey amounted to 55.6%. A total of 17.2% of Syrian males and 34.8% of females deemed illiterate and living outside the camps were included in the research [20]. Ultimately, 911 applicants who met the criteria were the target group for research. A questionnaire was applied to 598 (65.6%) of the 911 people who agreed to participate in the study, and questionnaires of 27 people were excluded from the analysis due to incomplete information. Finally, analyses were made on the questionnaires of 571 people (Figure 1).

A total of 6835 Syrian patients applied to the center where the research was conducted within 3 months and constitutes our population. With OpenEpi Version 3.01 software, the minimum number of people was calculated as 364 with 50% frequency and 5% margin of error. Anyone over the age of 18 who applied to the center and was literate was invited to the study, and the entire target population was reached (*n*: 571).

### 2.3. Survey Form

The questionnaire was prepared in Turkish by the researchers via literature scanning. Later, the questionnaire was translated into Arabic by a translator who speaks native Turkish but is also fluent in Arabic. A pilot study was conducted in the same center to evaluate the suitability of the questionnaire, such as meaning, expression, and content. The questionnaires of 20 people reached for the pilot application were not included in the study. The finalized questionnaire was re-evaluated by Syrian immigrants whose mother tongue was Arabic, and after it was found appropriate, data collection began.

The survey consisted of 3 sections. The first section included sociodemographic variables (e.g., age, gender, education status, employment status) and Turkish understanding/speaking status, the second section consisted of the COVID-19 vaccination status, and the last section included the THL-32 Health literacy scale.

### 2.4. THL-32 Scale

The THL-32 Scale was developed from the HLS-EU-Q47 (The European Health Literacy Survey Questionnaire). As a result of the reliability and validity study conducted, the matrix took its final form as eight components (accessing health-related information, understanding health-related information, evaluating health-related information, using/applying health-related information) with two dimensions (treatment and service and prevention of diseases/health promotion) and four processes. The general Cronbach Alpha coefficient of the scale was 0.927, the Cronbach Alpha coefficient of “Treatment and Service Sub-Dimension” was 0.880, and the “Dimension of Preventing Diseases and Promoting Health” was 0.863.

The scale can also be evaluated in 4 categories according to the scores obtained: 0–25 points are considered insufficient, >25–33 points as problematic/limited, >33–42 points as sufficient, and >42–50 points as perfect health literacy [21]. The Arabic version of the TSOY-32 scale used in the study was applied to Syrian immigrants living throughout the country in 2018 and 2020 within the scope of The SIHHAT Project (2020 Cronbach’s Alpha value: 0.980) [9]. The same scale was applied in another study examining the relationship between health behaviors and health literacy in Syrian immigrants, and Cronbach’s alpha value was found to be 0.953 [22]. Cronbach’s Alpha value, which indicates the internal consistency of the components of the Arabic version of the THL-32 Scale used in the study, was 0.978. The two main sub-dimensions, treatment and service index scores, and disease prevention and health promotion index scores also showed a high level of internal consistency (Cronbach’s Alpha values of 0.961 and 0.969, respectively).

### 2.5. Statistical Analysis

Descriptive statistics were presented as percentage, mean, and standard deviation values. The chi-square test was used for categorical variables and the Student *t*-test compared numerical variables to examine the differences between the groups. The internal consistency of the components of the THL-32 Arabic version was evaluated with Chronbach α.

Independent predictors of COVID-19 vaccination status were examined using logistic regression analysis, and *p* ≤ 0.05 was considered significant. Statistical analyses were performed in IBM SPSS Statistics 25.0 program.

## 3. Results

### 3.1. Sociodemographic Characteristics

A total of 598 people were reached, and 571 people were included in the analysis (27 surveys were excluded due to missing data). A total of 80.7% of the participants were women, the mean age was 31.92 ± 6.14, and the education level of 26.6% was high school. A total of 83.2% of the immigrants were married, and 80.7% had children. Those who understood but could not speak Turkish were 47.5%, and 79.9% had been residing in Turkey for more than five years. The average residency time was 6.14 ± 2.22. The data of the sociodemographic variables of the population are presented in Table 1.

### 3.2. COVID-19 Vaccination Status

Per the Centers for Disease Control and Prevention (CDC), the first two doses of COVID-19 vaccination are considered primary vaccination, and, accordingly, 33.5% of the immigrants in the study were vaccinated [23]. However, 55.0% did not have any COVID-19 vaccines.

Vaccination status according to some sociodemographic variables in the univariate analyses is summarized in Table 2. Accordingly, the vaccination rate was significantly higher in males, those aged 35 and over, those with an education level of high school and above, and those working in a regular job. Moreover, the rate of COVID-19 vaccination was higher in immigrants who evaluated their economic status as moderate and above those with any chronic disease.

### 3.3. Health Literacy

The health literacy level of only 1.1% of the immigrants was determined as “excellent.” Furthermore, 68.7% of them were at the level of “insufficient”, 20.7% of them were at “problematic/limited”, and 9.6% of them were at the level of “sufficient” health literacy. The mean general health literacy index score of the participants was 17.02 ± 12.53. The mean General Health Literacy Index score of men was 18.38 ± 13.76, and that of women was 16.70 ± 12.21 (*p* > 0.05) (Table 3).

**Figure 2:**
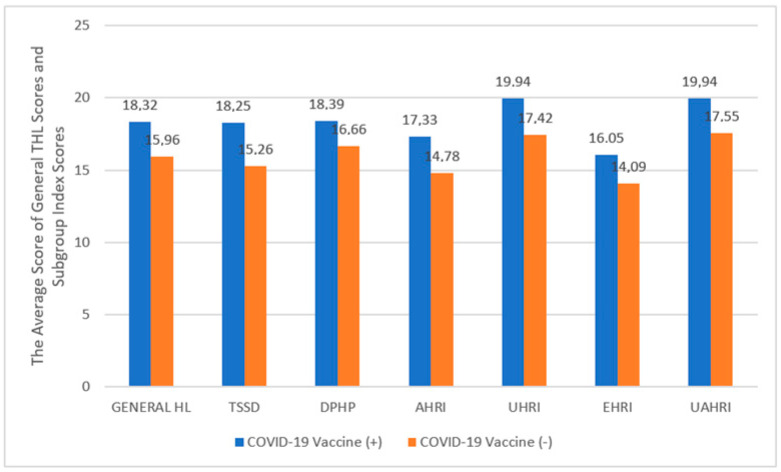
Average of General THL scores and Subgroup Index Scores according to the status of vaccination with the COVID-19 vaccine in immigrants, Istanbul, 2022. General HL: General Health Literacy (THL-32); TSSD: Treatment and Service Sub-Dimension; DPHP: Disease Prevention and Health Promotion; AHRI: Access to Health-Related Information; UHRI: Understanding Health-Related Information; EHRI: Evaluating Health-Related Information; UAHRI: Using/Application of Health-Related Information.

According to the THL-32 Scale scores, the general health literacy, treatment, service access to health-related information, and understanding and application of health-related information were higher for the vaccinated cohort compared to those who did not have the COVID-19 vaccine (*p*-values 0.025; 0.005; 0.017; 0.030; 0.036, respectively) (Figure 2).

A logistic regression model was created to evaluate the effect of some sociodemographic variables on getting the COVID-19 vaccine. Variables associated with COVID-19 vaccination status in the created model were being male (OR: 1.755; 95%CI: 1.133–2.718; *p* = 0.012), elder age (OR: 1.029; 95%CI: 1.01–1.048; *p* = 0.002), having a perception of middle and higher economic status (OR: 1.503; 95%CI: 1.029–2.197; *p* = 0.035), and having a chronic disease in the family (OR: 1.838, 95%CI: 1.297–2.604, *p* = 0.001). A high THL-32 Scale Treatment and Service sub-dimension index score was also associated with the model (OR: 1.017; 95%CI: 1.003–1.031; *p* = 0.016) (Table 4).

## 4. Discussion

In our study, the relationship between health literacy levels, socio-demographic characteristics, and participation in health screenings of immigrants who applied to the Expanded Migrant Health Center and their status of having the COVID-19 vaccine were examined. The health of people who had to migrate is affected by the severe conditions brought on by the migration conditions as well as the social determinants of health. This delicate situation can also negatively affect the protection and improvement of the health of immigrants suffering from low health literacy and their access to health services, such as chronic diseases and immunization [24].

According to our study results, almost half (45.0%) of immigrants had been vaccinated with at least one dose of the COVID-19 vaccine. Until June 2022, when our study data were collected, more than 15 million cases of COVID-19 and more than 99,000 deaths due to COVID-19 occurred in the whole society of Turkey [14]. Moreover, 93% of the population was vaccinated for at least one dose in the same period, and the primary vaccination rate was 85.5% [18]. The first vaccination in Turkey started in January 2021 in groups prioritized according to risky occupations, age, and chronic diseases. Subsequently, vaccination studies targeted the whole society, where access to all levels of health services can be achieved quickly. Furthermore, a free COVID-19 vaccine was administered at Immigrant Health Centers, as well as other centers, to facilitate access to the vaccine for the immigrant population and to increase vaccination [15,17]. Even though we are in a busy period of the pandemic and vaccination studies, and vaccinations are provided free of charge to everyone without any priority, more than half of the immigrants did not receive the COVID-19 vaccine. According to the results of a study conducted by the Red Crescent, an important social service organization in the country, with applicants to its centers in 15 provinces in 2021, it was stated that 71.1% of the immigrants were vaccinated at least one dose [17]. Vaccination rates vary in immigrant studies around the world. In a study conducted in Australia with a multinational refugee group (the majority of which were Arabic speakers), the vaccination rate was found to be 12% [25]. Similar to our country, a study in Norway determined that the probability of receiving the COVID-19 vaccine is one-third lower for immigrants than for non-immigrants [26]. There are other similar studies in which the probability of not being vaccinated in immigrants is 8–11 times higher than in the local population [27]. In a similar study conducted with elderly Syrian refugees in Lebanon, 42.5% of those who had at least one dose of the COVID-19 vaccine were below the local population [28]. Success in the epidemic can only be achieved with high vaccine coverage [29]. In a meta-analysis, vaccination acceptance of immigrants and refugees was found to be 73%. The fact that the majority of the studies included in the meta-analysis were from developed countries with a low refugee density may explain the high vaccine acceptance [30]. In a study investigating the factors affecting vaccination acceptance amongst immigrants in Jordan, it was found that almost 90% of the participants agreed to be vaccinated against COVID-19 and understood the importance of raising knowledge and awareness [31].

Achieving high vaccination rates in vulnerable groups such as immigrants (as well as the local population) is a critical point in epidemic management. The data of our study, in which we determined the low vaccination rate, was collected from a single center, serving only immigrants and foreign nationals. The majority of the applicants to the center were women who have a lower level of health literacy than men. Across the country, where easy and free access to the vaccine is provided for immigrants, and many studies are carried out by ministries, both professional and non-governmental organizations to increase knowledge and awareness, the rate of at least one dose of vaccination in the local population is above 90% but only 45% in immigrants. This is an issue that needs improvement. Increasing the quantitative studies on the factors affecting the acceptance of this population-specific vaccine and conducting qualitative studies may shed further light on the subject. Studies report that another critical factor affecting this is health literacy [32].

In our study, almost 90% of the immigrants were observed to have insufficient and problematic health literacy levels. In studies on the health literacy levels of immigrants in our country, insufficient and problematic health literacy levels were between 49.5% and 94.7% [9,10,22]. The general health literacy score and all subgroup (treatment and service, access to health-related information, understanding health-related information, and using and applying health-related information) index scores obtained from the THL-32 Scale of the immigrants who were vaccinated against COVID-19 were higher than those who did not have the vaccine. As the level of health literacy increases, participation in vaccination increases [33]. Our study also demonstrated that getting a higher score in the treatment and service sub-dimension was effective in getting the COVID-19 vaccine. The treatment and service sub-dimension is the individual’s ability to access, understand, evaluate, and use health-related information on medical issues. The fact that this score is high may have contributed to the increase in the acceptance of the newly developed COVID-19 vaccine and its application by individuals applying to institutions where they can access this service. Some studies provide evidence of a relationship between seasonal influenza vaccine intake and health literacy [34]. In a study conducted with immigrants in Jordan and in another study conducted with Syrian immigrants, the importance of knowledge and awareness regarding the COVID-19 vaccine is emphasized [28,31]. Health literacy has a direct impact on the individual’s ability to access sufficient information about health. Therefore, studies to increase health literacy will be beneficial, especially in interventions to vulnerable groups. It is known that health literacy also forms the basis for vaccine literacy. Therefore, interventions should be made to increase the level of health literacy for individuals to increase their participation in vaccination and to understand the information about the vaccine [32]. Some researchers have suggested that such interventions may help reduce inequalities in access to preventive health care [35]. In addition, the vaccination behavior of individuals’ physical accessibility (language, free vaccination, distance to vaccination site, working hours, etc.), understanding information about vaccination (understanding the severity of the disease and the benefit of vaccination, language-communication, health knowledge level, etc.), religious and cultural approaches to vaccination (especially the attitude of the immediate environment, life experiences in the pre-migration period, and the vaccination attitude of the country of origin), and upbringing can all be influential factors. In addition, factors such as the length of stay in the destination country, income status, education level, health literacy level, and trust in health services are also influential [32,35]. It should also be noted that the data of our study, in which we observed a lower vaccination rate, were collected only from a single center serving immigrants and foreign nationals, and that the majority of the applicants were women with a lower level of health literacy than men.

The World Health Organization considers factors such as nationality and legal status, linguistic and cultural differences, administrative barriers, and lack of information about health services as barriers to accessing health services [36]. In parallel with this, adult immigrants with different legal statuses, different languages, and different cultures have access to appropriate health information in the countries they migrated to, and often have difficulties in managing and understanding their health problems and highly complex health systems [10]. All these negativities in immigrants were accompanied by the COVID-19 pandemic, disease, anxiety, the problems brought on by the disease, and problems such as unemployment, malnutrition, access to limited hygiene materials, poor socioeconomic conditions, and poor housing as a result of the measures taken to prevent the disease [37]. As a result, inequalities have deepened amongst immigrants. Moreover, women are less vaccinated than men. There are studies in the literature with similar results [32,38].

Immigrant women are more socially isolated, do not go into the local population as much as men (as a result, language continues to be a barrier in the country they live in, too), and their lower education level may have played a role in their low health literacy. In our study, as with the results of many studies, it was determined that the probability of getting vaccinated increased as the age of the participants increased. Those with chronic diseases, those with chronic diseases in their families, and those with good economic status are also vaccinated more. In a modeling study conducted in Istanbul in the first year of the pandemic, the epidemic reproduction rate (R0) was higher at younger ages [39]. Although the pandemic reproduction rate is higher in younger ages, it was observed in studies conducted in our country and the world that the vaccine acceptance of individuals increases as age increases and as the economic situation improves [19,39,40]. Reasons such as the increased incidence of chronic diseases with age, the possibility of experiencing complications of COVID-19 at a higher age, more visits to healthcare services, and more frequent contact with healthcare professionals may all increase their awareness of and acceptance of vaccination.

In addition, our results showed that doctors were the first health information source of the participants during the epidemic period, with the Internet second. As a source of health information, people still rely primarily on healthcare providers. However, technological developments in recent years have enabled it to be used in different ways, such as social media and the internet, in order to transmit health information. Immigrants with language and communication problems may refer to the internet more frequently as a source of health information. The WHO recommends implementing digital health literacy for populations which use the Internet frequently. Informative brochures, videos, and trainings are all included on the website of the Ministry of Health of Turkey in their own language, making it easier for them to access accurate information [10,18].

### Strengths and Limitations

One of the strengths of this study is that it was conducted in the largest fortified migrant health center in Istanbul, the province with the highest number of refugees in Turkey. Fewer studies have been found on the factors affecting the acceptance of the COVID-19 vaccine in immigrants worldwide and in Turkey.

There are also some limitations of our study. First of all, the study could only be carried out in a single center during a period when the COVID-19 epidemic was intense. Therefore, applications were limited. This center mostly provides health services to Syrian immigrants, but it also serves many foreign nationals. In this respect, the results cannot be generalized to the center. In addition, the inclusion of illiterate immigrants is another limitation due to the scale we used. The fact that the study was conducted in a public center may have caused a perception of threat among immigrants and biased responses. The fact that the data were collected only on Mondays, Wednesdays, and Fridays may have contributed to the selection bias.

## 5. Conclusions

In this study, conducted with Syrians Under Temporary Protection in Istanbul, it was revealed that the level of health literacy is effective in vaccination recipients. Vaccination response was found to be higher in those with high health literacy levels.

Regarding the data, besides men, those with a family history of chronic diseases, those with good economic status, the elderly, and those with a high Treatment and Service Sub-Dimension index of the THL-32 Scale were more likely to have the COVID-19 vaccine.

### Future Implications

Studies to teach Turkish and advance the education level of Syrians will contribute to the increase in health literacy. Increasing health literacy proficiency will also increase determinative health effects. Therefore, treatment and compliance will increase with early diagnosis, and unnecessary hospital admissions and health costs will decrease as a result. Considering the long-term consequences of migration, advanced national and global action plans are needed.

## Figures and Tables

**Table 1:** Socio-demographic characteristics of immigrants by gender, Istanbul, 2022.

Socio-Demographic Variables		Male (*n*: 110)		Female (*n*: 461)		Total (*n*: 571)	
		*n*	%	*n*	%	*n*	%
Age groups	18–24	18	16.3	110	23.8	128	22.4
	25–34	51	46.4	210	45.6	261	45.7
	≥35	41	37.3	141	30.6	182	31.9
Educational status **	Literate	1	0.9	23	5.0	24	4.2
	Primary School	26	23.6	97	21.0	123	21.5
	Primary/Middle School	26	23.6	124	26.9	150	26.3
	High School	27	24.6	125	27.2	152	26.6
	University	22	20.0	90	19.5	112	19.6
	Master’s/PhD	8	7.3	2	0.4	10	1.8
Marital status **	Single	28	25.5	26	5.6	54	9.5
	Married	79	71.8	396	85.9	475	83.2
	Widow/Divorced/Separated	3	2.7	39	8.5	42	7.3
Childbearing status **	Yes	69	62.7	392	85.0	461	80.7
	No	41	37.3	69	15.0	110	19.3
Duration of residence in Turkey	0–4 years	26	23.6	89	19.3	115	20.1
	5–8 years	72	65.5	315	68.3	387	67.8
	9–12 years	12	10.9	57	12.4	69	12.1
Ability to speak and understand Turkish **	Understands and speaks	48	43.6	100	21.7	148	25.9
	Understands but cannot speak	53	48.2	218	47.3	271	47.5
	Does not understand and cannot speak	9	8.2	143	31.0	152	26.6
Employment status **	Has a regular job	28	25.5	7	1.5	35	6.1
	Has a temporary job	58	52.7	42	9.1	100	17.5
	Unemployed	24	21.8	412	89.4	436	76.4
Perception of the economic situation	Very Bad/Bad	31	28.2	140	30.4	171	29.9
	Moderate/Good/Very good	79	71.8	321	69.6	400	70.1
Chronic disease status	Yes	28	25.5	113	24.5	141	24.7
	No	82	74.5	348	75.5	430	75.3
Chronic disease status in the family	Yes	46	41.8	211	45.8	257	45.0
	No	64	58.2	250	54.2	314	55.0
Health perception	Very Bad/Bad	11	10.0	47	10.2	58	10.2
	Moderate	39	35.5	201	43.6	240	42.0
	Good/Very good	60	54.5	213	46.2	273	47.8

** *p* < 0.05.

**Table 2:** Vaccination status according to socio-demographic variables, Istanbul, 2022.

		COVID-19 Vaccine (+)	COVID-19 Vaccine (−)	*p*
		*n* (%)	*n* (%)	
Gender	Male	62 (56.4)	48(43.6)	0.008
	Female	195 (42.3)	266 (57.7)	
Age group	18–24	44 (34.4)	84 (65.6)	0.002
	25–34	114 (43.7)	147(56.3)	
	35 years and over	99 (54.4)	83 (45.6)	
Marital status	Single	21 (38.9)	33 (61.1)	0.535
	Married	215 (45.3)	260 (54.7)	
	Widow/Divorced/Separated	21 (50.0)	21 (50.0)	
Educational status	Under high school	120 (40.4)	177 (59.6)	0.021
	High school and above	137 (50.0)	137 (50.0)	
Child status	Yes	210 (45.6)	251 (54.4)	0.592
	No	47 (42.7)	63 (57.3)	
Employment status	Has a regular job	24 (68.6)	11 (31.4)	0.005
	Working in temporary jobs	50 (50.0)	50 (50.0)	
	Unemployed	183 (42.0)	253 (58.0)	
Perception of the economic situation	Very Bad/Bad	65 (38.0)	106 (62.0)	0.028
	Moderate and Above	192 (48.0)	208 (52.0)	
Duration of residence in Turkey	0–4 years	51 (44.3)	64 (55.7)	0.964
	5–8	174 (45.0)	213 (55.0)	
	9–12	32 (46.4)	37 (53.6)	
Ability to speak and understand Turkish	I understand and speak	70 (47.3)	78 (52.7)	0.276
	I understand but can not speak	127 (45.9)	144 (53.1)	
	I do not understand and I cannot speak	60 (39.5)	92 (60.5)	
Chronic disease status	Yes	77 (54.6)	64 (45.4)	0.008
	No	180 (41.9)	250 (58.1)	
Chronic disease status in the family	Yes	139 (54.1)	118 (45.9)	<0.001
	No	118 (37.6)	196 (62.4)	
Health perception	Very Bad/Bad	27 (46.6)	31 (53.4)	0.389
	Moderate	100 (41.7)	140 (58.3)	
	Good/Very good	130 (47.6)	143 (52.4)	

**Table 3:** Immigrants’ THL-32 Scale score and sub-group score averages, Istanbul, 2022.

Health Literacy Subgroup	Index Mean (SD)	Health Literacy Level (%)			
		Insufficient	Problematic/Limited	Sufficient	Excellent
General Health Literacy	17.0 (12.5)	68.7	20.7	9.6	1.1
TSSD	16.6 (12.6)	70.4	17.9	11.6	0.2
DPHP	17.4 (13.8)	65.5	16.5	15.8	2.3
AHRI	15.9 (12.7)	72.5	14.0	12.8	0.7
UHRI	18.5 (13.8)	62.9	15.6	19.3	2.3
EHRI	14.9 (12.3)	76.9	12.3	10.2	0.7
UAHRI	18.6 (13.5)	62.9	17.5	18.0	1.6

TSSD: Treatment and Service Sub-Dimension; DPHP: Disease Prevention and Health Promotion; AHRI: Access to Health-Related Information; UHRI: Understanding Health-Related Information; EHRI: Evaluating Health-Related Information; UAHRI: Using/Application of Health-Related Information.

**Table 4:** Independent determinants of immigrants’ COVID-19 vaccination status, logistic regression model, Istanbul, 2022.

		COVID-19 Vaccine		
		OR	95% CI	*p*
Gender	Male	1.755	1.133–2.718	0.012
	Female			
Age		1.029	1.011–1.048	0.002
Economical status	Very Bad/Bad			0.035
	Moderate/Good/Very good	1.503	1.029–2.197	
Chronic Disease Status in Family	None			0.001
	Yes	1.838	1.297–2.604	
THL Treatment and Service Sub-Dimension Index Score		1.017	1.003–1.031	0.016
Constant		0.124		<0.001

## Data Availability

Not applicable.
